# Breaking up with ATM

**Published:** 2018-01-18

**Authors:** Marek Adamowicz

**Affiliations:** 1 Genome Damage and Stability Centre, School of Life Sciences, University of Sussex, Falmer, Brighton BN1 9RH, UK

## Abstract

ATM kinase is a master regulator of the DNA damage response (DDR). A recently published report from the d’Adda di Fagagna laboratory^1^ sheds a light onto our understanding of ATM activation. In this short-commentary we will expand on this and other work to perceive better some of the aspects of ATM regulation.

## ATM and DNA damage response activation

**Figure 1:**
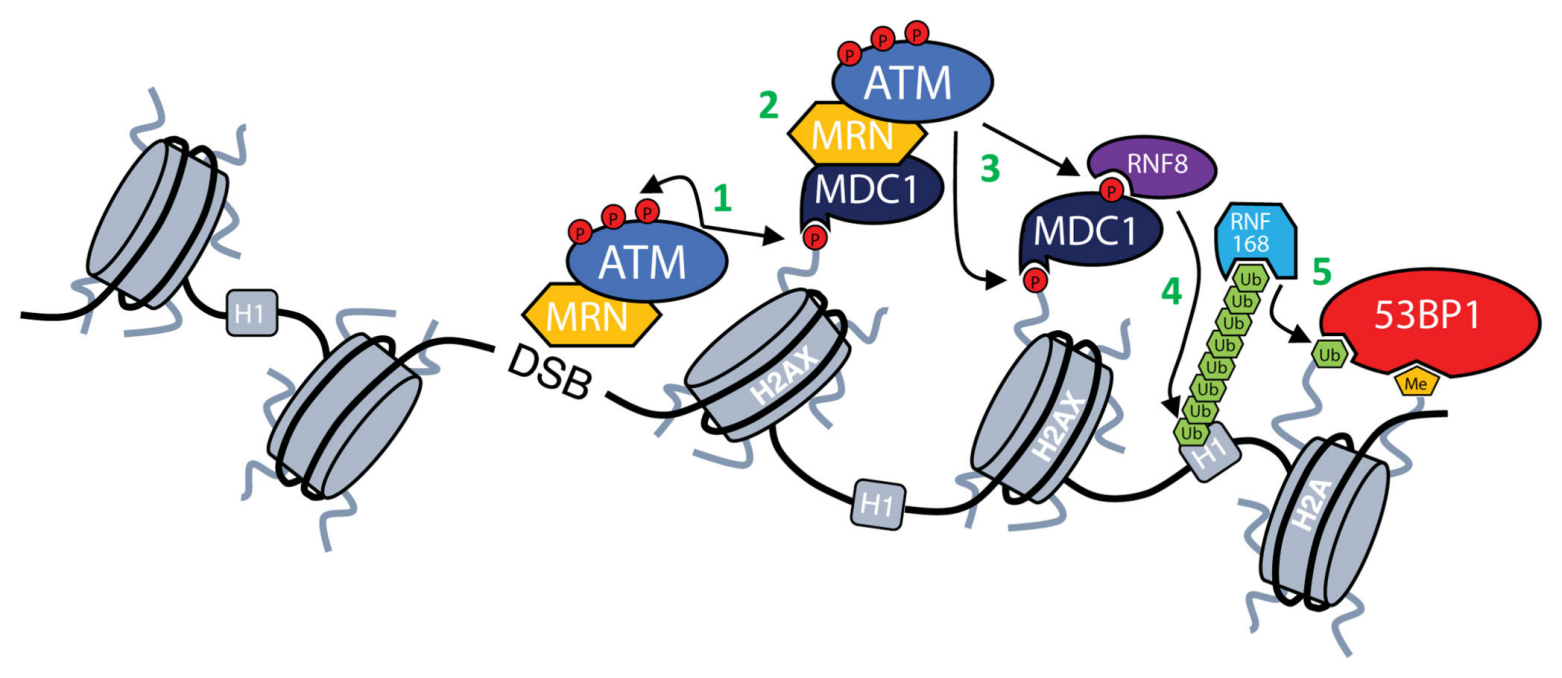
DNA damage response cascade. First step in the DDR activation is recruitment of ATM kinase by MRN complex to DSBs through its interaction with NBS1^4^,^5^. At the DSB ATM is activated (for details see Figure 2) and phosphorylates both itself and other substrates (**1**). One of the most important substrates of ATM is H2AX^6^, which provides a scaffold for the further accumulation of DDR factors^7^. γH2AX is recognized and bound by MDC1 that then enables further accumulation of ATM-MRN complex and spreading of γH2AX around DSBs (**2**)^8^,^9^. ATM-mediated phosphorylation of MDC1 allows also for the recruitment of RNF8 ubiquitin ligase (**3**), which ubiquitinates histone H1 and enables the recruitment of another ubiquitin ligase RNF168 (**4**)^10^,^11^, which in turn ubiquitinates histone H2A^12^. Ubiquitination of H2A together with the deposition of methyl group done by MMSET^13^ results in the recruitment of 53BP1 (**5**), which coordinates with other factors DNA repair pathway choice^14^.

ATM belongs to PI3K-like kinase family that consists of ATR, DNA-PKcs, mTOR, TRRAP and SMG1 kinases, which share similar structures and ability to phosphorylate their substrates on Serine and Threonine residues^2^. ATM was first discovered and cloned over 20 years ago in the laboratory of Yossi Shiloh^3^. ATM is recruited to DNA double strand breaks (DSBs) by MRN complex, composed of MRE11, RAD50 and NBS1 proteins, which senses DNA damage and quickly localizes to the DSBs. This process induces ATM activation and triggers a downstream cascade that leads to the engagement of DDR factors like: MDC1 and 53BP1, around DNA lesions (Figure 1).

**Figure 2:**
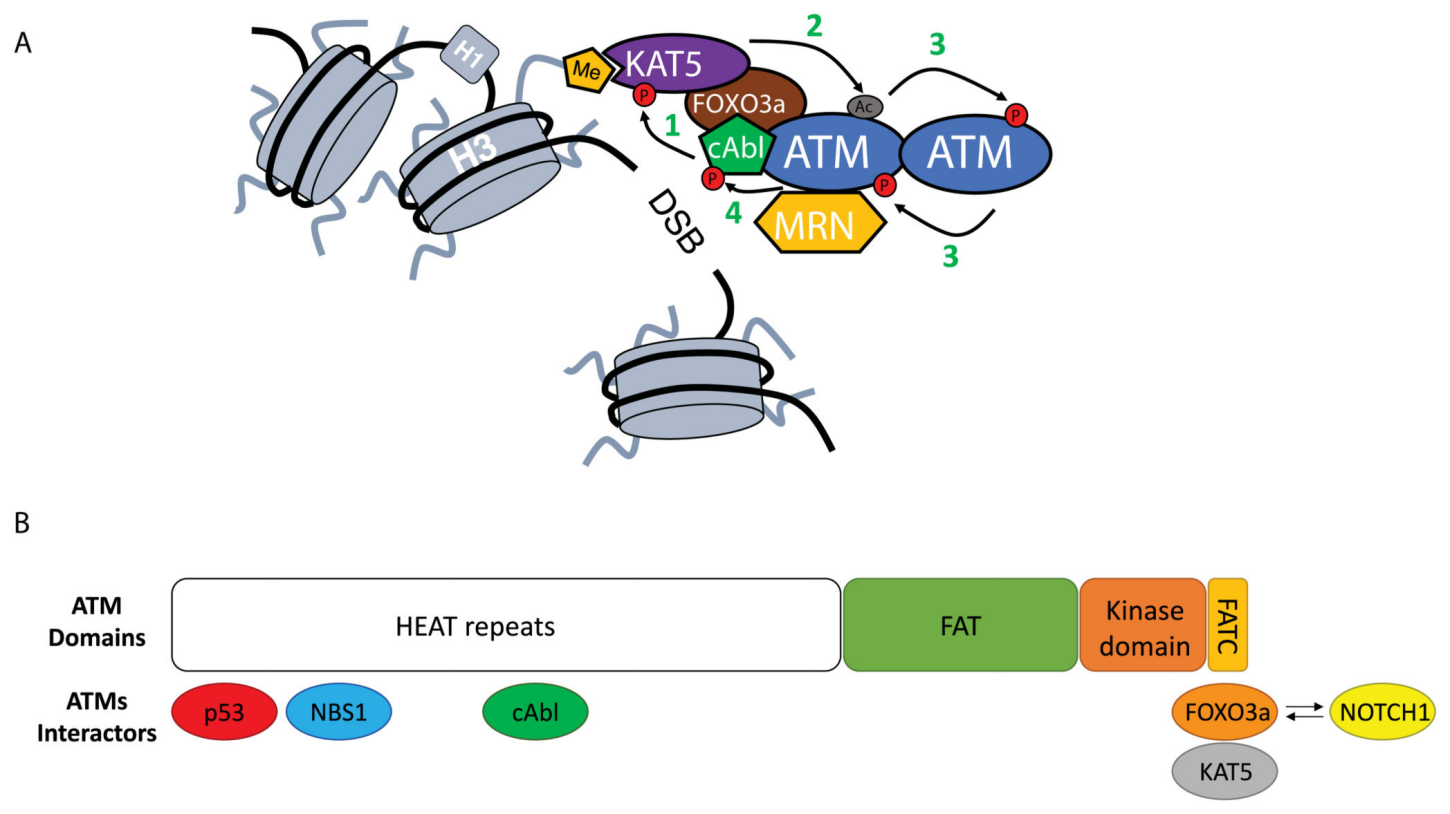
Close-up on ATM activation. A) Upon MRN-mediated recruitment to DSBs ATM undergoes an activation process. First, cAbl kinase phosphorylates KAT5 on Tyrosine 44 (**1**)^18^, which together with KAT5 interaction with H3K9m3 leads to KAT5 activation^19^. This way stimulated KAT5 will mediate acetylation of ATM in its PRD domain (Lysine 3016)(**2**)^15^,^20^. As a consequence of acetylation, ATM undergoes structural changes in its dimer form that allows for the phosphorylation in trans each of ATM monomers (**3**)^16^,^20^. ATM autophosphorylation leads to its monomerization and full activation^16^. At the end, activated ATM will generate a positive feedback loop and phosphorylate cAbl kinase leading to its increased activation (**4**)^21^. B) Structure of the ATM kinase (1-3056aa; Domains: FAT 1966–2566aa; kinase domain 2614–2960aa and FATC 3025–3056aa) together with its most important interactors and their binding sites^1^,^22^–^25^. Competition between FOXO3a and NOTCH1 for the binding to the FATC domain is depicted with arrows.

Upon recruitment to DSBs, ATM dimer undergoes a complicated process of activation. First, it is acetylated by lysine acetyltransferase 5 (KAT5/TIP60) at Lysine 3016^15^, which is followed by autophosphorylation, in-trans on Serine 1981, leading to its monomerization and conferring access of ATM substrates to the kinase domain^16^,^17^(Figure 2A).

In recent work, Adamowicz et al., show that ATM localizes to the DSBs in a protein complex together with FOXO3a and KAT5/Tip60 (from now on referred to as AAC (ATM Activation Complex)). Formation of this complex is dependent on FOXO3a bridging an interaction between ATM and KAT5 (Figure 2A and B). Formation of AAC is inhibited by a well-known transcription factor, NOTCH^1^, which is a negative regulator of ATM activation through its ATM binding ability^24^ (Figure 2B). Interestingly this leads to MRN-mediated recruitment of ATM to DSBs without the latter being activated at the site of the damage, as observed by the lack of autophosphorylation (pSer1891ATM)^1^. Strikingly, although all downstream DDR events are attenuated due to NOTCH1-mediated ATM inactivation (including ATM-mediated phosphorylations like: p53, CHK2, SMC1), phosphorylation of the ATM main substrate, histone H2AX (Ser139H2AX, from now on referred as γH2AX) is not affected^1^,^24^. Below I will try to focus on those two aspects of ATM activation (ATM autophosphorylation and phosphorylation of H2AX), putting them in the context of already published reports. At the end, I will speculate about the role of NOTCH-FOXO3a competition in cells and possible future areas of ATM research.

## ATM phosphorylation – is it still a thing?

ATM phosphorylation on Serine 1981 is commonly used as a marker for activated ATM. ATM phosphorylation was first observed almost 20 years ago^26^,^27^ and since then many of ATM’s autophosphorylation sites have been identified^28^,^29^. It has been established that ATM autophosphorylation on Serine 1981 is necessary for ATM monomerization and kinase activation 16, 20. Additionally it has been shown that ATM mutants carrying the S1981A substitution do not rescue radio-sensitivity of the A-T cells in transfection experiments^16^. In agreement with those reports we have shown that NOTCH1-mediated inhibition of ATM autophosporylation resulted in the attenuation of phosphorylation of ATM downstream substrates and checkpoint impairment^1^,^24^. On the other hand it has been reported that mouse Atm S1987A mutants (mouse equivalent of human S1981A) are proficient in DDR and ATM kinase activity^30^. Moreover, it seems that other Atm autophosphorylation sites (S367 and S1893) are dispensable for Atm activation^31^. Interestingly in the *in vitro* kinase assay ATM S1981A mutant can phosphorylate CHK2 and p53 in response to linear dsDNA to the same extent as the wild-type ATM^32^. Although we observed NOTCH1 inhibiting ATM-mediated p53 phosphorylation *in vitro*^24^, in the light of the new results demonstrating disruption of KAT5 and FOXO3a from the AAC^1^ it would be interesting to see if addition of: KAT5, FOXO3a, H3K9m3 and cAbl would give similar results in an *in vitro* ATM kinase assay in the presence of NOTCH1 and mutated ATM (S1981A).

It has been suggested that ATM autophosphorylation is necessary for ATM retention at the DSBs^33^ because the ATM S1981A mutant, although recruited normally to DSBs, was not stabilized properly at the site of the damage^33^. In contrast we have reported that although NOTCH1 inhibited ATM autophosphorylation it did not affect ATM’s retention at the DSBs^1^. To understand these apparently contradictory observations one should look not just at the ATM autophosphorylation. We have previously reported that NOTCH1 inhibits kinase activity of ATM^24^, therefore it would be better to compare our results with the reports describing recruitment of the ATM kinase dead mutant (ATM KD). Indeed it has been shown that ATM KD mutant (that cannot undergo autophosphorylation) is recruited to the DSBs without any impairment of retention^34^,^35^. This is similar to our observation of the NOTCH1-mediated impact on ATM recruitment and autophosphorylation. Additionally, it was reported that cells carrying mutations in the KU70 and MRE11 nuclease actively recruit ATM to the DSBs (without retention impairment); although ATM does not undergo autophosphorylation in those conditions^36^. Overall, our results^1^,^24^, together with above mentioned reports, show our still incomplete understanding of ATM autophosporylation and its role in the ATM activity and DDR, that needs further elucidation.

## Meeting γH2AX at the FATC end

We have repeatedly observed that NOTCH1 inhibits ATM activation by blocking its autophosphorylation (Ser1981) and phosphorylation of downstream substrates such as KAP1, SMC1, p53, DNA-PKcs or CHK2^1^,^24^(Adamowicz et al – in press). Additionally, we have observed in our initial study using *Xenopus laevis* egg extract that NOTCH1 blocked a substantial amount of ATM-mediated phosphorylation^24^. However, of all observed NOTCH1-mediated ATM phosphorylation defects, phosphorylation of ATM’s main substrate, H2AX, remained unaffected.

It has been suggested that there is redundancy between PI3K-like kinases in terms of H2AX phosphorylation. DNA-PKcs or ATR kinases have been shown to phosphorylate H2AX^37^,^38^. Additionally, it has been reported that H2AX is phosphorylated to the same extent in the ATM WT and KO cells^37^,^39^. Indeed, experiments carried out in our laboratory showed that NOTCH1 neither blocks ATR nor DNA-PKcs kinase activity (Adamowicz et al – in press). However unexpectedly, when we performed analysis of the γH2AX foci formation in NOTCH1-expressing cells in the presence of either DNA-PKcs or ATR inhibitors we did not observe any difference in the H2AX phosphorylation (data unpublished – data available upon request).

These results suggest that H2AX can be phosphorylated by protein kinases other than ATM, ATR and DNA-PKcs. Indeed, it has been reported that JNK and p38 can phosphorylate H2AX in response of UV light irradiation or starvation respectively^40^,^41^. Additionally, it has been reported that VRK1 kinase can phosphorylate H2AX in response to IR in parallel to ATM kinase^42^. Moreover, VRK1 was shown to be necessary for the accumulation of DDR factors around DSBs, which implies more complex role of VRK1 in the DDR that needs further elucidation^43^.

Because NOTCH1, unlike small molecule ATM inhibitors, cannot directly inhibit ATM kinase activity we can speculate that NOTCH1 binding to ATM could strongly impair ATM substrate recognition, resulting in an inhibition of phosphorylation of some substrates such as p53 or CHK2, but not H2AX. It has been already reported that NOTCH1 can bind and hence modulate the substrate recognition of LSD1 demethylase^44^. Therefore, it can be possible that by binding to the FATC domain of ATM, NOTCH1 would strongly impair substrate recognition of ATM. Interestingly, it has been shown in yeast that deletion of last 10 amino acids (aa) of the FATC domain can impair Tel1 phosphorylation of Rad53, which was connected with the loss in its ability to interact with MRX complex^45^. Moreover, MRN complex was shown to help ATM in the substrate recognition by stimulating ATM binding to its substrates like p53 or CHK2^46^. On the other hand it has been published that in human cells deletion of last 10aa of the FATC domain of ATM does not lead to the impairment of ATM MRN-mediated response, but rather its ability to activate upon oxidative stress^47^. We have shown that although NOTCH1 binds to the FATC domain it does not affect interaction between ATM and MRN complex^1^. This suggests that if by binding to the ATM FATC domain NOTCH1 is perturbing ATM substrate recognition this effect is rather mediated by inhibition of KAT5-mediated acetylation. Impairment of ATM acetylation will then block structural changes in ATM that would lead to its monomerization and activation, inhibiting this way release of the ATM kinase domain otherwise hindered inside of its dimer structure^17^.

## Taking ATM down a NOTCH

NOTCH1 was very early connected to the tumorogenesis and marked as an oncogene due to its ability to induce tumour growth^48^. Activating mutations in NOTCH1 are present in many T-cell acute lymphoblastic leukemias (T-ALL)^49^ or breast cancers^50^. Indeed, we have found that NOTCH1 expression was negatively correlated with the ATM activation in the human breast cancer patients^24^. At the same time, other group reported that ectopic expression of NOTCH1 in cancer cells lead to their increased resistance to DNA damage in vivo^51^. Those data show that expression of high levels of NOTCH1 (due to activating mutations or ectopic expression) stimulate radioresistance and survival in cancer cells, resulting probably from the inhibition of p53-mediated apoptosis. Additionally, increased levels of NOTCH1 induce faster proliferation^52^ leading to replication stress. Although we showed that NOTCH1 inhibits ATM activation, this is not true for ATR kinase (Adamowicz et al – in press) resulting in the protection of NOTCH1-driven cancers from replication stress.

Neural stem cells (NSC) are known to express moderate levels of activated NOTCH1, which is necessary for their proliferation^53^. Interestingly it has been reported that induction of DDR in NSC leads to their spontaneous differentiation to astrocytes, which is dependent on ATM activation^54^. It is therefore possible to speculate that NOTCH1-mediated downregulation of ATM activation could tip the balance allowing for DNA damage repair without inducing differentiation. It is important to remember that physiological levels of NOTCH1 are low as compared to those observed in T-ALL cells or those achieved by ectopic expression, therefore observed effects of NOTCH1 activation might be very mild. Additionally, observed results might be an outcome of many different factors impacting at the same time on DDR. Indeed, it has been shown that SALL4 transcription factor expressed in stem cells favours ATM activation by its binding to MRN complex^55^.

In summary, I would like to propose that the physiological role of NOTCH1 is not to inhibit fully ATM activation, but rather to induce its mild impairment, to modulate a balance between the amount of DNA damage and DDR signalling. This would result in the suppression of DNA damage induced apoptosis or differentiation, giving time for necessary repair.

## There is plenty more ATM in the sea

**Figure 3:**
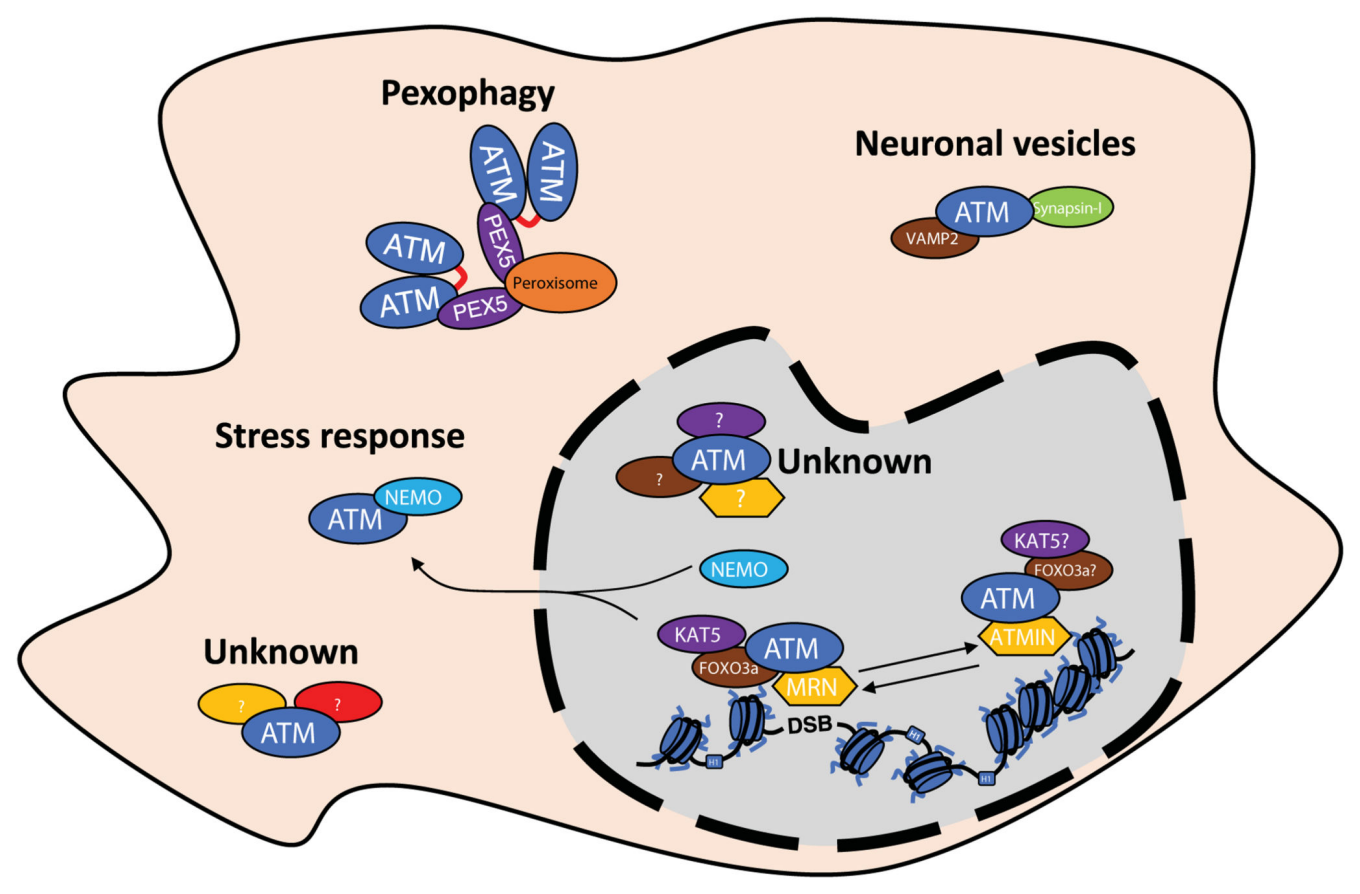
Existence of different ATM complexes in the cells. Schematic representation of the existence of different ATM complexes both in the nucleus as well as in the cytosol. For details please look into the text.

Formation of AAC is necessary for ATM activation at DSBs and DDR^1^,^24^. Apart from AAC, ATM relies also on MRN complex, which allows proper AAC localization and substrate recognition. Interestingly, in the nucleus, ATM has been described to exist in two different complexes. In has been shown that there is competition between MRN complex and ATMIN for binding to ATM (Figure 3)^56^,^57^. Studies have found that while MRN complex guides ATM in the response to DSBs, ATMIN is necessary during oxidative and hypotonic stresses^58^,^59^. It is possible that like MRN, ATMIN by binding to ATM regulates it substrate recognition and therefore its kinase activity in response to different stimuli. It would be interesting to see if the structure of AAC is preserved while complexed with ATMIN, and if so how it is involved in the ATM activation (Figure 3).

We tend to think about ATM through its role in DDR regulation, although ATM has been described to be involved in many more cellular processes like: stress response^60^, neuronal signal transmission^61^ or pexophagy^62^,^63^(Figure 3), which in every case requires its presence outside the nucleus. This implies that ATM is not always in complex with KAT5 or MRN complex. The presence of ATM in peroxisomes is a result of its interaction with PEX5, which is responsible for ATM peroxisome localization. In peroxisomes, ATM is activated by reactive oxygen species and formation of an active dimer, allowing ATM to control peroxisome phagocytosis^63^. The involvement of ATM in the stress response is connected to its interaction with NEMO and with shuttling between nucleus and cytosol^60^. Additionally, ATM has been described to localize in the cytosol of neuronal cells^64^ and has been implicated in the neuronal signal transmission by its interaction with VAMP2 and Synapsin-I^61^. It would be interesting to see if NOTCH1, which has very strong affinity to the FATC domain of ATM^1^, could be used as a tool for identifying new regulatory components of ATM complexes in the cytosol.

It is thought-provoking to picture ATM in different complexes that differentially regulate its activity and substrate recognition. The identification of different active ATM complexes opens new and exciting areas of research and raises even more fascinating questions. For example, how is ATM activation in those complexes stimulated, and how is ATM substrate recognition and kinase activity regulated? Hopefully, in the near future we will know the answers.
